# Influence of arginine vasopressin on the ultradian dynamics of Hypothalamic-Pituitary-Adrenal axis

**DOI:** 10.3389/fendo.2022.976323

**Published:** 2022-10-05

**Authors:** Aleksandra S. Stojiljković, Željko Čupić, Stevan Maćešić, Ana Ivanović-Šašić, Ljiljana Kolar-Anić

**Affiliations:** ^1^ Institute of General and Physical Chemistry, University of Belgrade, Belgrade, Serbia; ^2^ Institute of Chemistry, Technology and Metallurgy, National Institute of the Republic of Serbia, University of Belgrade, Belgrade, Serbia; ^3^ Faculty of Physical Chemistry, University of Belgrade, Belgrade, Serbia

**Keywords:** Hypothalamic-Pituitary-Adrenal (HPA) axis, arginine vasopressin, AVP and CRH synergy, HPA ultradian dynamics, stoichiometric modeling, numerical simulations

## Abstract

Numerous studies on humans and animals have indicated that the corticotrophin-releasing hormone (CRH) and arginine vasopressin (AVP) stimulate both individually and synergistically secretion of adrenocorticotropic hormone (ACTH) by corticotropic cells in anterior pituitary. With aim to characterize and better comprehend the mechanisms underlying the effects of AVP on Hypothalamic-Pituitary-Adrenal (HPA) axis ultradian dynamics, AVP is here incorporated into our previously proposed stoichiometric model of HPA axis in humans. This extended nonlinear network reaction model took into account AVP by: reaction steps associated with two separate inflows of AVP into pituitary portal system, that is synthesized and released from hypothalamic parvocellular and magnocellular neuronal populations, as well as summarized reaction steps related to its individual and synergistic action with CRH on corticotropic cells. To explore the properties of extended model and its capacity to emulate the effects of AVP, nonlinear dynamical systems theory and bifurcation analyses based on numerical simulations were utilized to determine the dependence of ultradian oscillations on rate constants of the inflows of CRH and AVP from parvocellular neuronal populations, the conditions under which dynamical transitions occur due to their synergistic action and, moreover, the types of these transitions. The results show that under certain conditions, HPA system could enter into oscillatory dynamic states from stable steady state and vice versa under the influence of synergy reaction rate constant. Transitions between these dynamical states were always through supercritical Andronov-Hopf bifurcation point. Also, results revealed the conditions under which amplitudes of ultradian oscillations could increase several-fold due to CRH and AVP synergistic stimulation of ACTH secretion in accordance with results reported in the literature. Moreover, results showed experimentally observed superiority of CRH as a stimulator of ACTH secretion compared to AVP in humans. The proposed model can be very useful in studies related to the role of AVP and its synergistic action with CRH in life-threatening circumstances such as acute homeostasis dynamic crisis, autoimmune inflammations or severe hypovolemia requiring instant or several-days sustained corticosteroid excess levels. Moreover, the model can be helpful for investigations of indirect AVP-induced HPA activity by exogenously administered AVP used in therapeutic treatment.

## Introduction

The interplay between the functions of hypothalamus, pituitary and adrenal glands creates a complex nonlinear neuroendocrine system known as the hypothalamic-pituitary-adrenal (HPA) axis. The HPA axis activity is necessary for maintaining homeostasis under physiologically normal and various stressful conditions through the action of its main hormones. Their actions are expressed through complex biochemical transformations that are intertwined *via* positive and negative feedback loops. Complex interplay between these feedback mechanisms and coupling of the HPA axis with circadian clock system give rise to daily rhythm of the main HPA axis hormones characterized by ultradian oscillations (caused by internal feedbacks) superimposed on circadian oscillations (greatly influenced by external cycles) (1–3). In humans, periods of ultradian oscillations of the HPA axis hormones altogether range between 20 minutes to 2 hours, while periods of their circadian oscillations are around 24 hours (1, 2, 4–9). Disruption in regulation of HPA axis oscillatory dynamics can lead to the development of many psychiatric and metabolic diseases (2, 10). Thus, additional experimental and theoretical investigations have been conducted to examine all constituents and detailed dynamic regulatory mechanisms underlying the HPA axis function under basal physiological and pathophysiological conditions. Effects of acute and chronic exposure to various endogenous/exogenous stressors on the HPA axis dynamics and its impairment in disease states have been also largely studied.

Beside corticotropin-releasing hormone (CRH), as one of main HPA hormones, many other neuroactive substances have been found to enter the pituitary portal circulation. This includes somatostatin, neurotensin, angiotensin II, enkephalin, arginine vasopressin (AVP), dopamine and others (11–27). However, AVP has received the most attention among them so far, due to its relevant role and effects on corticotrope cells’ function and HPA axis activity. Namely, it has been indicated that both CRH and AVP act on their own receptors (28–32) on corticotrope cells in anterior pituitary to stimulate adrenocorticotropic hormone (ACTH) production and release with different (26, 33–41) or even similar potencies (42). Moreover, CRH and AVP are also capable of potentiating each other’s activity (37, 38). Their synergistic stimulation of ACTH production and secretion by corticotrope cells could yield a several-fold higher output than due to the action of either one individually (26, 34–41, 43–47). However, the exact mechanism underlying their individual and synergistic action is yet to be clarified. In essence, CRH and AVP are both required as stimulatory inputs to corticotrope cells for a complete ACTH response in physiologically normal as well as in various stressful conditions (32, 38, 48–51). The ACTH stimulates the steroidogenesis from cholesterol in adrenal gland and secretion of steroid hormones. Principal representative of steroid hormones in humans is cortisol (CORT), which exerts its effect on most tissues in the body and largely regulates the activity of the HPA system through its feedback mechanisms (2, 3).

Theoretical studies of effects of AVP alone and in synergy with CRH on HPA axis activity and dynamics are very scarce. To our best knowledge, in only two theoretical studies (52, 53), impacts of AVP and/or CRH on corticotrope cells’ activity are encompassed in mathematical models. These models are non-stoichiometric and describe: synthesis, accumulations, secretions and concentrations time series of CRH and AVP, ACTH and CORT as well as nonlinear feedback mechanisms with time delays considerations and effects of CRH and AVP on corticotrope cells, each by suitable mathematical function. These functions known as the ones that formally can produce oscillatory dynamics cannot be obtained as results of any real reaction mechanism comprised of a set of chemical reactions. On the other hand, if stoichiometric network approach is applied, complex biochemical transformations can be concisely described by a simplified network of interactions between the considered biochemical species. The rates of these transformations strictly follow the law of mass action. Using this approach, HPA axis oscillatory dynamics in model would emerge due to the nonlinearity of underlying biochemical interactions of considered species through positive and negative feedback mechanisms. Moreover, its global behavior would be tractable to mathematical analysis.

In this study, our previously proposed stoichiometric model of HPA axis in humans (54) has been extended to emulate the influence of AVP on ACTH production and secretion. Using such extended HPA axis model, we investigated the AVP and CRH effects and synergistic influence on ultradian dynamics. For that purpose, numerical simulations and bifurcation analysis have been employed.

## Model description

Stoichiometric model of HPA axis activity in humans developed in our earlier work (54) has been used as the basis to incorporate influence of arginine vasopressin (AVP) into the HPA mechanism described by the initial model. The AVP was introduced into this initial model of HPA axis activity by five new reaction steps ((R2.2), (R4), (R5.2), (R5.3) and (R12)), which are highlighted in bold in the **Table 1**. Reaction steps presented in **Table 1** describe net reactions of a series of complex biochemical processes. Accordingly, reaction steps (R2.2) and (R4) describe appropriate inflows of AVP into the pituitary portal circulation as net reactions of a series of processes of AVP biosynthesis and release from the parvocellular part of the paraventricular nucleus (PVN), and from the magnocellular neurosecretory system of the hypothalamus, respectively (32, 48–51, 55). In essence, reaction steps (R2.1) and (R2.2) depict inflows of CRH and AVP, respectively, from neuronal populations of the same part of PVN. On the other hand, inflow of AVP from the magnocellular neurons (R4) can be much more abundant (up to 10-fold) than from parvocellular neurons under normal condition (51). Furthermore, these neurons are not under prominent negative feedback control by adrenal corticosteroids (51). The AVP originating from both of these parvocellular and magnocellular neuronal populations enter the pituitary portal circulation and regulate the pituitary production and secretion of ACTH, as it is summarized by reaction step (R5.2) (32, 48–51, 55). Reaction step (R5.3) describes net reaction of a series of complex biochemical processes of ACTH production and secretion by corticotrope cells that is also stimulated by CRH and AVP acting synergistically (32, 48–51, 55). End-result of complex biochemical processes leading to the elimination of AVP is described by reaction step (R12). The remaining reaction steps in **Table 1** are related to the initial model of HPA axis in humans and are described in ref. (54).

**Table 1:** The extended model of the HPA axis activity in humans with incorporated arginine vasopressin (AVP) as an additional dynamic variable; reaction steps associated with AVP are given in bold.

$\overset{k_{1}}{\to }CHOL$	k_1_ = 1.38 × 10^−4^ mol dm^−3^ min^−1^	(R1)
$\overset{k_{2.1}}{\to }CRH$	k_2.1_ = 1.83 × 10^−8^ mol dm^−3^ min^−1^	(R2.1)
$\overset{k_{2.2}}{\to }AVP$	k_2.2_ = 1.83 × 10^−8^ mol dm^−3^ min^−1^	**(R2.2)**
$\overset{k_{3}}{\to }ALDO$	k_3_ = 6.09 × 10^−11^ mol dm^−3^ min^−1^	(R3)
$\overset{k_{4}}{\to }AVP$	k_4_ = 1.537 × 10^−9^ mol dm^−3^ min^−1^	**(R4)**
$CRH\overset{k_{5.1}}{\to }ACTH$	k_5.1_ = 1.83 × 10^4^ min^−1^	(R5.1)
$AVP\overset{k_{5.2}}{\to }ACTH$	k_5.2_ = 7.79 × 10^−3^ min^−1^	**(R5.2)**
$CRH+AVP\overset{k_{5.3}}{\to }ACTH$	k_5.3_ = 3.66 × 10^2^ mol^−1^ dm^3^ min^−1^	**(R5.3)**
$CHOL+ACTH\overset{k_{6}}{\to }CORT$	k_6_ = 11.94 mol^−1^ dm^3^ min^−1^	(R6)
$CHOL+ACTH\overset{k_{7}}{\to }ALDO$	k_7_ = 9.552 × 10^−2^ mol^−1^ dm^3^ min^−1^	(R7)
$ACTH+2CORT\overset{k_{8}}{\to }3CORT$	k_8_ = 1.26 × 10^14^ mol^−2^ dm^6^ min^−1^	(R8)
$ALDO+2CORT\overset{k_{9}}{\to }CORT$	k_9_ = 7.05 × 10^12^ mol^−2^ dm^6^ min^−1^	(R9)
$CHOL\overset{k_{10}}{\to }P_{1}$	k_10_ = 4.5 × 10^−2^ min^−1^	(R10)
$CRH\overset{k_{11}}{\to }P_{2}$	k_11_ = 1.1 × 10^−1^ min^−1^	(R11)
$AVP\overset{k_{12}}{\to }P_{3}$	k_12_ = 1.386 × 10^−1^ min^−1^	**(R12)**
$ACTH\overset{k_{13}}{\to }P_{4}$	k_13_ = 5.35 × 10^−2^ min^−1^	(R13)
$CORT\overset{k_{14}}{\to }P_{5}$	k_14_ = 4.1 × 10^−1^ min^−1^	(R14)
$ALDO\overset{k_{15}}{\to }P_{6}$	k_15_ = 1.35 × 10^−1^ min^−1^	(R15)

The extended model given in **Table 1** is now comprised of 18 reaction steps and six independent dynamic variables representing concentrations of cholesterol ([CHOL]), CRH ([CRH]), AVP ([AVP]), ACTH ([ACTH]), CORT ([CORT]) and ALDO ([ALDO])). Corresponding kinetic rate constants are labeled by k_a_, a = 1 **-** 15.

Temporal concentration evolutions of all species are described by a system of ordinary differential equations (ODE) obtained by applying the law of mass action on reaction steps shown in **Table 1**:


(1) $$\frac{d[CHOL]}{dt}=k_{1}-(k_{6}+k_{7})[CHOL][ACTH]-k_{10}[CHOL]$$

(2) $$\frac{d[CRH]}{dt}=k_{2.1}-(k_{5.1}+k_{11})[CRH]-k_{5.3}[CRH][AVP]$$

(3) $$\frac{d[AVP]}{dt}=k_{2.2}+k_{4}-k_{5.2}[AVP]-k_{5.3}[CRH][AVP]-k_{12}[AVP]$$

(4) $$\begin{array}{l} \frac{d[ACTH]}{dt}=k_{5.1}[CRH]+k_{5.2}[AVP]+k_{5.3}[CRH][AVP]\\ \;\;\;-(k_{6}+k_{7})[CHOL][ACTH]\\ \;\;\;-k_{8}[ACTH][CORT{]}^{2}-k_{13}[ACTH] \end{array}$$

(5) $$\begin{array}{ll} \frac{d[CORT]}{dt}=k_{6}[CHOL][ACTH]+k_{8}[ACTH][CORT{]}^{2}-k_{9}[ALDO][CORT{]}^{2} & -k_{14}[CORT] \end{array}$$

(6) $$\frac{d[ALDO]}{dt}=k_{3}+k_{7}[CHOL][ACTH]-k_{9}[ALDO]{\left[CORT\right]}^{2}-k_{15}[ALDO]$$

## Methods

### Numerical method

In order to solve ODE describing the temporal evolution of extended model (**Table 1**), numerical simulations were conducted in the Matlab software package. Ode15s solver routine based on the Gear algorithm (56) for integration of stiff differential equations was used. In all simulations, the absolute and relative tolerance errors were 1 × 10^−20^ and 3 × 10^−12^, respectively. Integrations of the model with 1 × 10^−14^, 5 × 10^−14^ and 1 × 10^−15^ absolute tolerance levels were also tested. It was noticed that only with absolute tolerance error of 1 × 10^−15^, numerical simulations were stable. Yet, in some investigated cases, 1 × 10^−20^ absolute tolerance level was necessary and therefore chosen as the final value. The initial concentrations in numerical simulations were: [CHOL]_0_ = 3.4 × 10^−4^ mol dm^−3^, [CRH]_0_ = 1 × 10^−12^ mol dm^−3^, [AVP]_0_ = 1 × 10^−12^ mol dm^−3^, [ACTH]_0_ = 8 × 10^−8^ mol dm^−3^, [CORT]_0_ = 4 × 10^−8^ mol dm^−3^ and [ALDO]_0_ = 1.5 × 10^−9^ mol dm^−3^. If not otherwise stated, rate constants (k_a_, a = 1 - 15) used in the numerical simulations were the same as in **Table 1**. Whenever possible, values of rate constant of reaction steps related to the initial model (not in bold in **Table 1**) were the same as in our previous papers (57, 58). Values of the rate constants of reaction steps (R2.2), (R4), (R5.2), (R5.3) and (R12), associated with AVP effects were derived from (39, 54, 59, 60) unless otherwise specified.

### Bifurcation analysis

To determine boundaries of oscillatory domain as a function of reaction rate constants of the reactions associated with CRH (R2.1) and AVP inflows (R2.2) (**Table 1**), the bifurcation analysis based on numerical continuation method was applied. Namely, during each numerical continuation, selected value of k_2.1_ in the range between 1 × 10^−9^ and 2 × 10^−8^ mol dm^−3^ min^−1^ was kept constant while the value of k_2.2_ was varied to find the region where ultradian oscillatory dynamics can be obtained. For each value of k_2.2_ steady-state concentrations were evaluated together with stability of considered steady state. All other rate constants had values as indicated in **Table 1**. By this method of bifurcation analysis (Method 1a, **Supplementary material**), the boundaries of the oscillatory domain were obtained as a function of k_2.1_ and k_2.2_ for one fixed k_5.3_ value given in **Table 1**. Afterwards, the impact of CRH and AVP acting synergistically on HPA oscillatory dynamics (R5.3) (**Table 1**) was investigated by another method of bifurcation analysis (Method 1b, **Supplementary material**). This was achieved by applying numerical continuation with reaction rate constant k_5.3_ as continuation parameter, for couple of k_2.1_ and k_2.2_ values that are selected to be very close to boundaries of oscillatory domain identified by Method 1a. By this method, for each k_5.3_ value the steady-state concentrations were evaluated together with stability of the considered steady-state. Once more, all other rate constants had values as indicated in **Table 1** unless otherwise specified.

The ultradian dynamics of HPA system for various combinations of k_2.1_ and k_2.2_ values, including the ones around oscillatory domain boundaries, was also examined by bifurcation analysis based on numerical simulations of dynamic states obtained by the above defined differential equations (1) - (6) (Method 2, **Supplementary material**). Namely, for a given couple of k_2.1_ and k_2.2_ values, set of numerical simulations were performed as a function of various values of rate constant k_5.3_. By this method, bifurcation diagrams with k_5.3_ as the control parameter were formed. The stable steady state of the system was denoted by one point in the [CORT] - k_5.3_ bifurcation diagram whereas the oscillatory state was denoted by two points corresponding to [CORT] values in oscillation minimum and maximum. In the latter case, the steady state is unstable, and hence, unattainable to numerical simulations. These bifurcation diagrams were obtained by varying k_5.3_ values in the range between 1.098 × 10^3^ and 1.098 × 10^20^ mol^−1^ dm^3^ min^−1^. If values of k_5.3_ < 1.098 × 10^3^ mol^−1^ dm^3^ min^−1^ or values of k_5.3_ > 1.098 × 10^20^ mol^−1^ dm^3^ min^−1^ were applied, no significant dynamic changes were observed. Furthermore, by the same method, the diagrams of amplitudes of [CORT] oscillations as a function of k_5.3_ were also analyzed. All other rate constants had values as indicated in **Table 1** unless otherwise specified.

## Results

**Figure 1:**
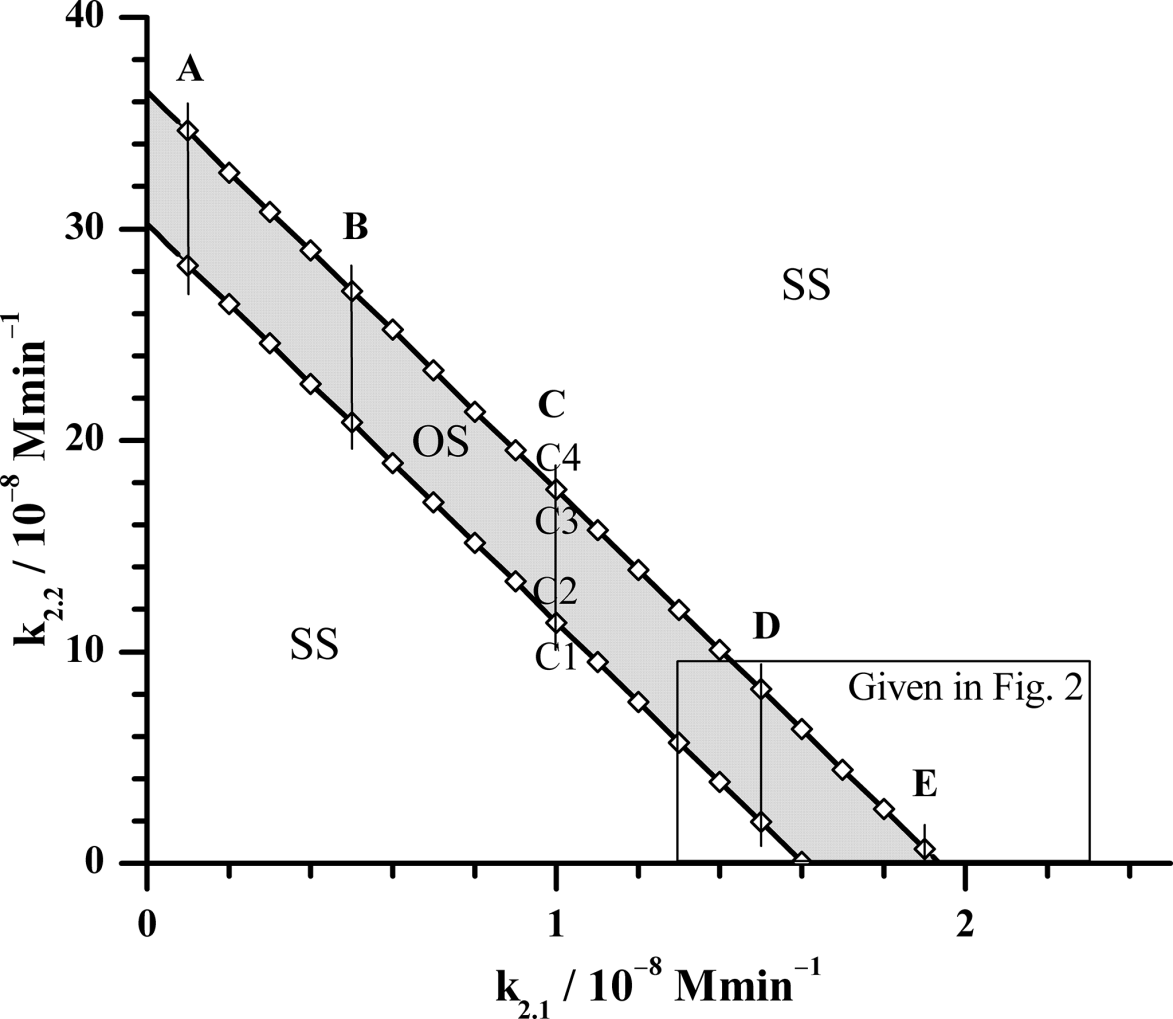
Position of supercritical AH bifurcation as a function of rate constants k_2.1_ and k_2.2_, obtained by Method 1a of bifurcation analysis for k_5.3_ = 3.66 × 10^2^ mol^−1^ dm^3^ min^−1^ (the value given in **Table 1**). The calculated AH bifurcations, depicted by open rhombuses (◊) are interconnected by solid lines for better visualization of the oscillatory domain. OS - oscillatory domain; SS - stable steady states. Five fixed k_2.1_ values denoted as cases: A (k_2.1_ = 0.1 × 10^−8^ mol dm^−3^ min^−1^), B (k_2.1_ = 0.5 × 10^−8^ mol dm^−3^ min^−1^), C (k_2.1_ = 1.0 × 10^−8^ mol dm^−3^ min^−1^), D (k_2.1_ = 1.5 × 10^−8^ mol dm^−3^ min^−1^) and E (k_2.1_ = 1.9 × 10^−8^ mol dm^−3^ min^−1^) for which Method 1b was applied near bifurcation points. In the case C, Method 1b was applied in points where values of rate constant k_2.2_ are: C1 (k_2.2_ = 11.2 × 10^−8^ mol dm^−3^ min^−1^), C2 (k_2.2_ = 11.4 × 10^−8^ mol dm^−3^ min^−1^), C3 (k_2.2_ = 17.6 × 10^−8^ mol dm^−3^ min^−1^) and C4 (k_2.2_ = 18.6 × 10^−8^ mol dm^−3^ min^−1^) (More details will be shown in **Figure 3**). All other rate constants used in both Method 1a and 1b analysis had values as presented in **Table 1**, except in Method 1b where k_5.3_ was varied.

**Figure 2:**
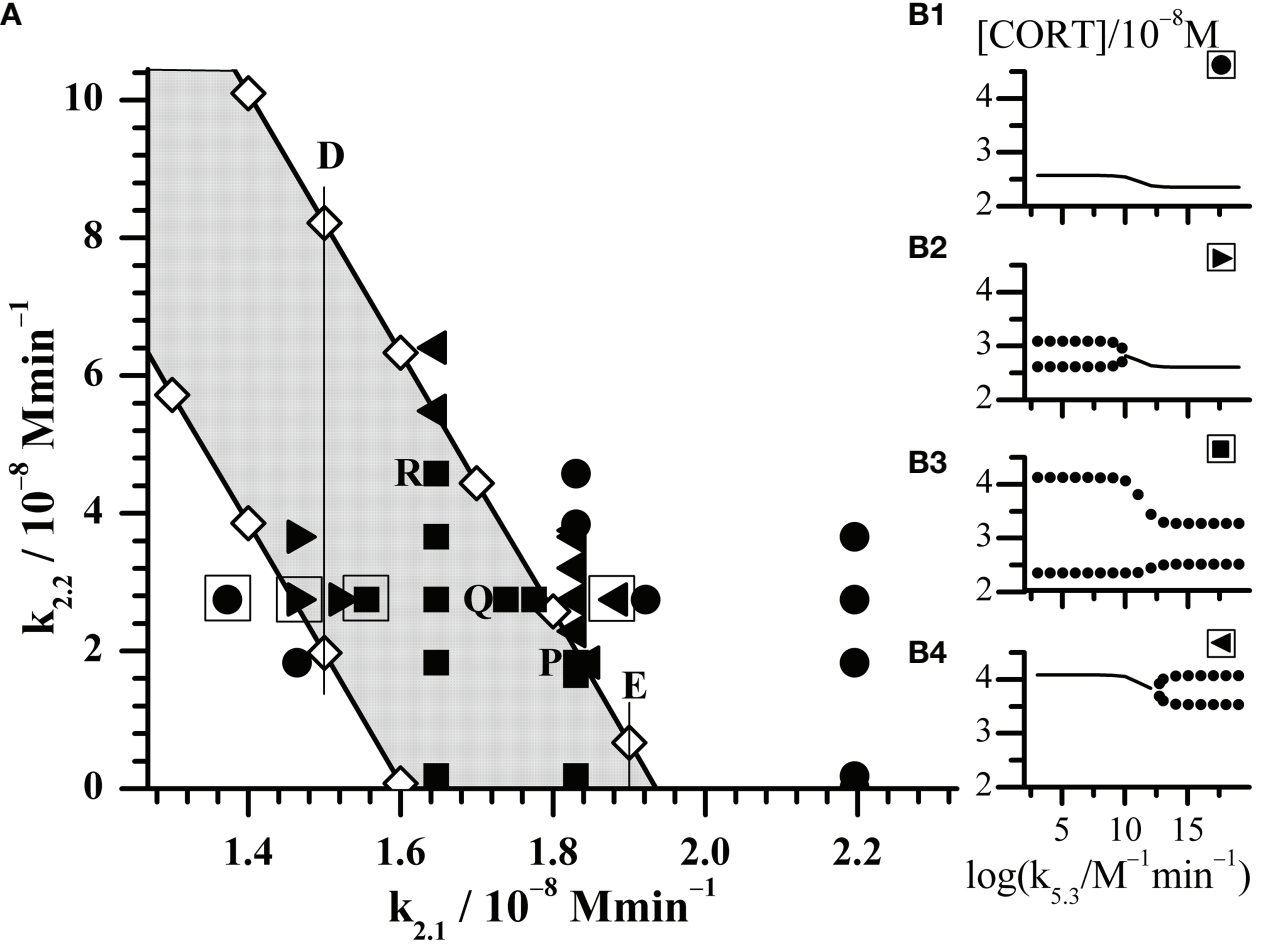
Enlarged part of the bifurcation diagram framed by rectangle in **Figure 1**. **(A)** Black symbols represent dynamic states identified by Method 2 with increasing k_5.3_ as control parameter. Circles (**●**) depict points where only stable steady states were found; right triangles (►) depict points where transition from oscillatory states into stable steady states was observed; squares (◼) depict points where only oscillatory states were observed; left triangles (◄) depict points where transition from stable steady states into oscillatory states was observed. **(B1–B4)** the outlooks of bifurcation diagrams depicted by corresponding black symbols in the square-bordered examples. The selected points P, Q and R will be discussed in **Figure 4**.

Using the above explained methods, the oscillatory dynamics as an essential characteristic of the HPA axis is particularly examined. Therefore, selection of the conditions that lead to its emergence is of great importance for model optimization. Since the goal of our investigations was to examine how AVP alone and in synergy with CRH can modify ultradian dynamics of HPA axis, the starting point in our analysis was to determine the dependence of boundaries of the oscillatory domain on the inflows of CRH and AVP from the parvocellular neuronal populations of PVN. These inflows represented by reactions (R2.1) and (R2.2) in **Table 1**, are initial steps of two parallel reaction pathways guided by signaling through CRH and AVP, respectively. They are interconnected through a complex feedback loops and capable to compensate each other. Therefore, the bifurcation analysis could provide new insights into how various levels of these two hormones’ concentrations originating from the same inflow source affect ultradian dynamics of HPA model. This was achieved, here, by utilizing Method 1a of bifurcation analysis for k_5.3_ = 3.66 × 10^2^ mol^−1^ dm^3^ min^−1^ (**Table 1**). Results in **Figure 1** show that oscillatory domain is confined between two straight lines which represent dependence of supercritical Andronov-Hopf (AH) bifurcation on k_2.1_ and k_2.2_. It could also be noticed that a small decrease in the value of k_2.1_ requires a much larger (circa by an order of magnitude) increase in the values of k_2.2_ in order to keep HPA model in the oscillatory regime. This suggests the inferiority of AVP compared to CRH as stimulator of ACTH secretion. However, if with increasing k_2.2_ at the same time k_2.1_ decreases so that the system approaches the left end of the oscillatory domain intersecting the y-axis in **Figure 1**, the values of k_2.2_ might become large enough to retain the system in oscillatory dynamic states, even though the value of k_2.1_ → 0 and so does its impact. Also, in this part of **Figure 1**, the value of k_4_, which was kept constant in this analysis (**Table 1**), has weak to no-significant influence compared to k_2.2_. In other words, in the vicinity of y-axis, the AVP reaction pathway governed by reaction (R2.2) (**Table 1**) tends to predominate over the CRH reaction pathway. On the other hand, with decrease of k_2.2_ and increase of k_2.1_ towards the right end of the oscillatory domain intersecting the x-axis, the impact of k_2.2_ may become comparable with k_4_ (1.537 × 10^−9^ mol dm^−3^ min^−1^, **Table 1**) and with its further decreases, more and more inferior and finally non-significant. In this part of **Figure 1**, also given at the bottom of **Figure 2**, the influence of k_4_ is generally the highest possible, but lower than the influence of k_2.1_ though. In essence, in the vicinity of x-axis as k_2.2_ → 0, CRH reaction pathway governed by reaction (R2.1) tends to prevail over the AVP reaction pathway, in contrast to the left end of oscillatory domain.

**Figure 3:**
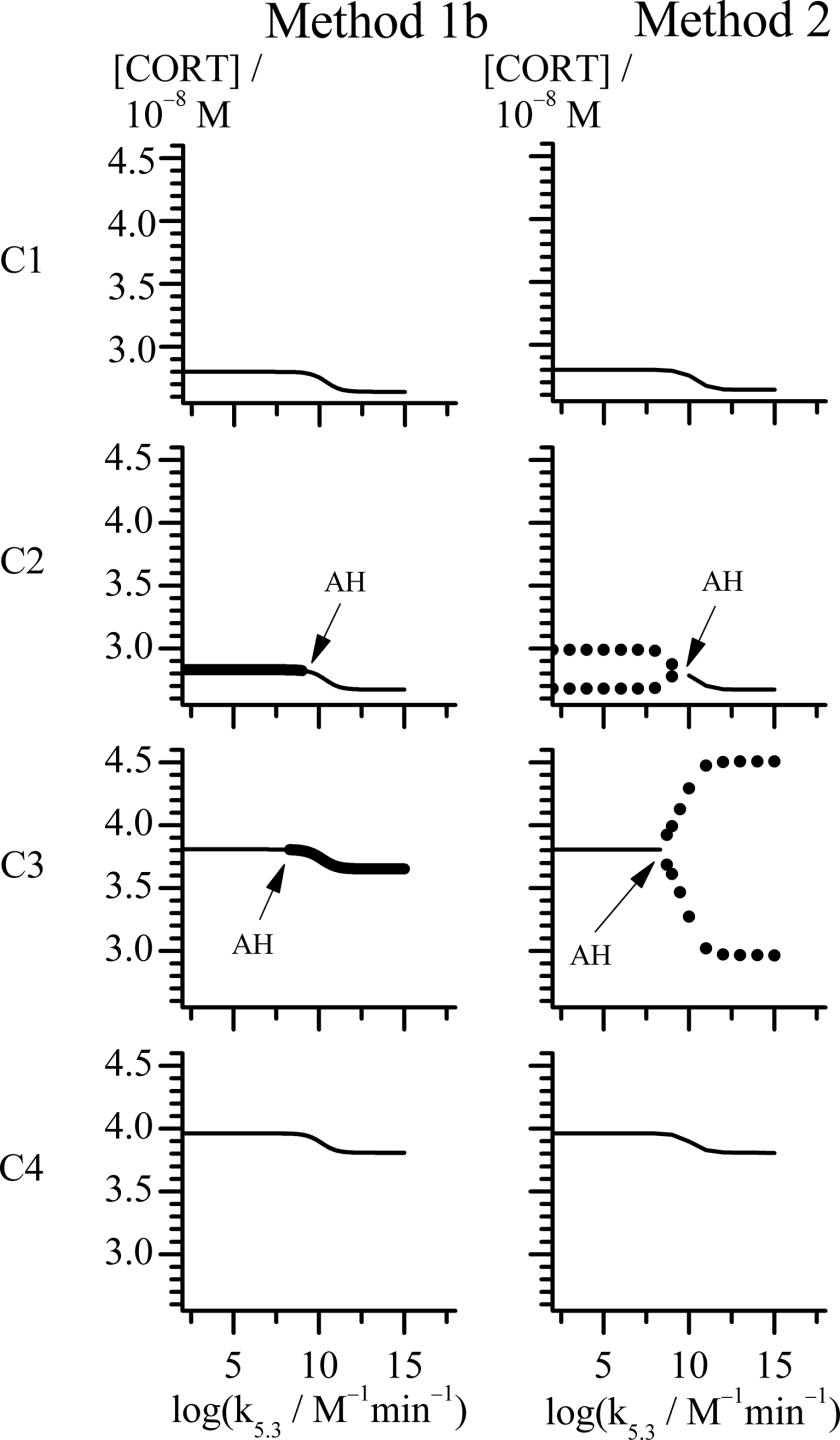
Bifurcation diagrams obtained with rate constant k_5.3_ as control parameter by Methods 1b and 2 (see points C1-C4 in **Figure 1**). All points in case C share the same value of k_2.1_ = 1 × 10^−8^ mol dm^−3^ min^−1^, while for each point: C1 (k_2.2_ = 11.2 × 10^−8^ mol dm^−3^ min^−1^), C2 (k_2.2_ = 11.4 × 10^−8^ mol dm^−3^ min^−1^), C3 (k_2.2_ = 17.6 × 10^−8^ mol dm^−3^ min^−1^) and C4 (k_2.2_ = 18.6 × 10^−8^ mol dm^−3^ min^−1^). Thin line represents stable steady state (Method 1b and 2). Unstable steady states are represented by thick line (Method 1b). Minimums and maximums in oscillations of cortisol concentrations are represented by circles (Method 2). All other rate constants used in analysis had values as presented in **Table 1**.

**Figure 4:**
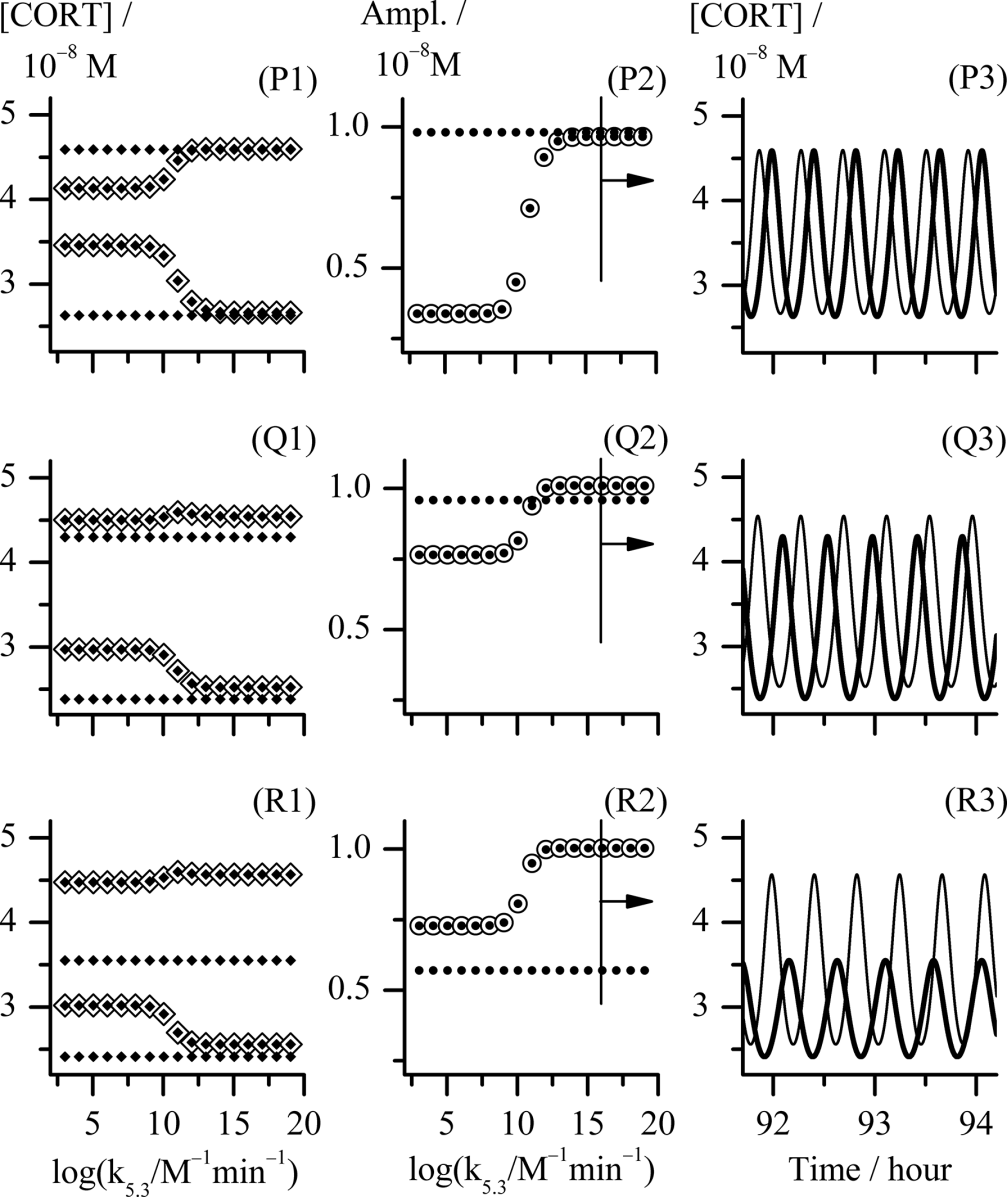
Analysis of three cases of combination of k_2.1_ and k_2.2_ reaction rate constants values corresponding to the points P, Q and R in **Figure 2**. Point P, k_2.1_ = k_2.2_ = 1.830 × 10^−8^ mol dm^−3^ min^−1^; point Q, (k_2.1_ and k_2.2_) = (1.738 and 2.745) × 10^−8^ mol dm^−3^ min^−1^, respectively; point R, (k_2.1_ and k_2.2_) = (1.647 and 4.575) × 10^−8^ mol dm^−3^ min^−1^, respectively; (P1) - (R1) bifurcation diagrams and (P2) - (R2) diagrams of change of [CORT] oscillation amplitudes each obtained with k_5.3_ as control parameter using Method 2; (P3) - (R3) temporal evolutions of [CORT] for k_5.3_ = 1.098 × 10^16^ mol^−1^ dm^3^ min^−1^, for the arbitrarily chosen time interval between around 92 and 94 hours. All results referring to the extended HPA model are depicted by ◈, ⦿ and thinner curves in diagrams (P1) - (R1), (P2) - (R2) and (P3) - (R3), respectively. All results referring to the initial HPA axis model (54) in diagrams (P1) - (R1), (P2) - (R2) and (P3) - (R3) are depicted by ♦, **●** and thicker curves, respectively. In (P1) - (R1) bifurcation diagrams, [CORT] maximum and [CORT] minimum are denoted as pair of ◈ and pair of ♦ related to corresponding extended and initial HPA model, respectively. All other rate constants had values as given in **Table 1**.

**Figure 5:**
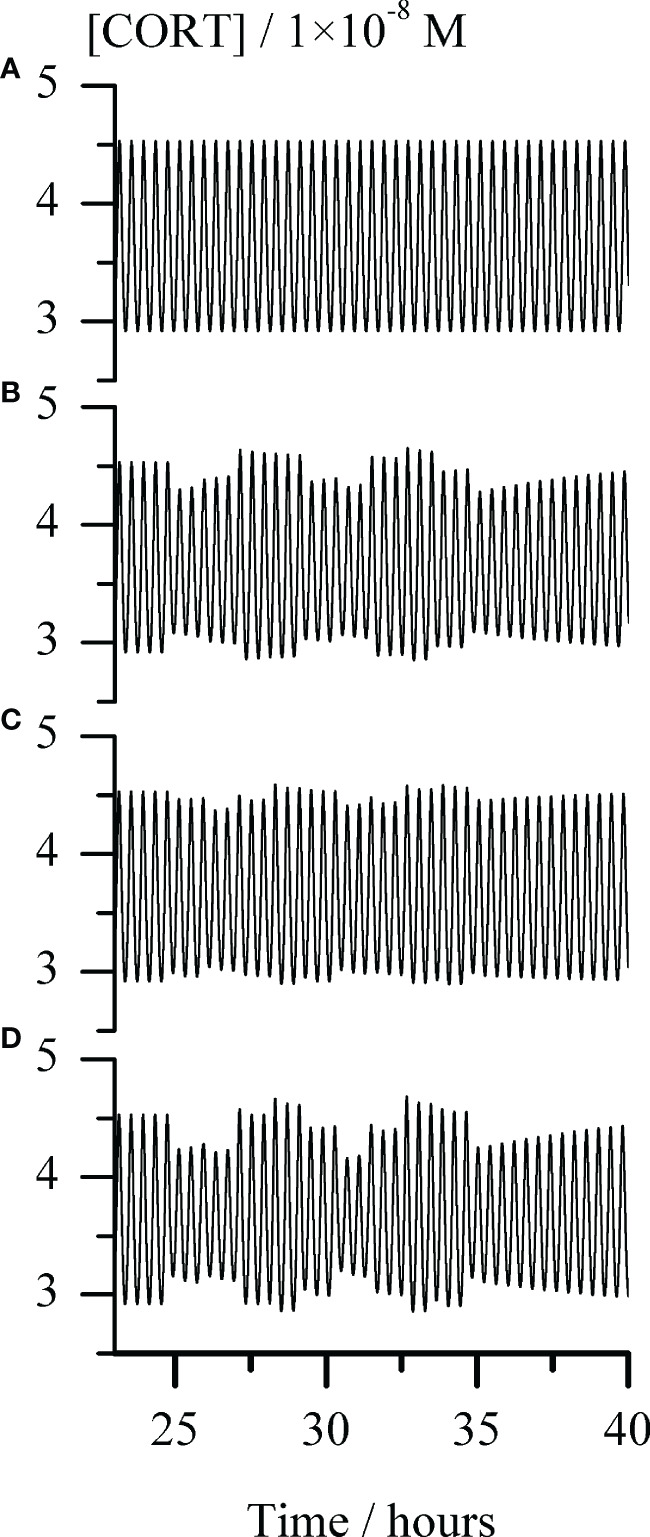
Temporal evolution of cortisol concentration ([CORT]) for repeating single-pulse perturbations with each stimulator of ACTH release alone and in combination, for the arbitrarily chosen time interval between around 23 and 40 hours. Graph **(A)** - the unperturbed extended model of HPA axis; Graphs **(B–D)** responses of the HPA axis to repeating pulse perturbations with: **(B)** CRH **(C)** AVP and **(D)** conjoint CRH and AVP. The perturbation intensities of each stimulator of ACTH secretion were the same in all corresponding cases, [CRH] = 2.5 × 10^−9^ M and AVP [AVP] = 50.0 × 10^−9^ M. In all Graphs **(B–D)**, the first pulse perturbations were applied in the maximum-to-minimum inflection point of a selected ultradian cortisol oscillation starting from the one at 1490^th^ minute (circa 24.8 h) and repeated every 66 minutes, giving a total of 10 pulses perturbations. In all cases presented in Graphs **(A–D)**, rate constants (k_2.1_ and k_2.2_) = (1.647 and 4.575) × 10^−8^ mol dm^−3^ min^−1^ and k_5.3_ = 1.098 × 10^10^ mol^−1^ dm^3^ min^−1^, while all other rate constants had values as given in **Table 1**.

With aim to compare the behavior of the here extended model with the initial model behavior (54, 61), the additional bifurcation analysis was done in some specific parts of bifurcation diagram depicted in **Figure 1**. First, detailed analysis of dynamic states was performed within the rectangle area shown in the bottom right part of **Figure 1**. The obtained results are given in **Figure 2**; **Tables 2**, **3**. Second, more general behavior of the extended model was analyzed for selected values of k_2.1_, marked by vertical lines A, B, C, D and E in **Figure 1**. More precisely, for each given k_2.1_ value two k_2.2_ values very close to each AH point were examined. Corresponding results are summarized in **Figure 3** for the case of C line (points C1 – C4), although similar behavior was detected for other values of k_2.1_ (A, B, D and E in **Figure 1**). Moreover, for three selected points in **Figure 2** (P, Q and R) yielding the particularly convenient forms of bifurcation diagrams, the two models of HPA axis activity (the initial and here extended ones) were compared to each other in order to further correlate the results of simulations obtained by Method 2 with experimental findings (39, 44–47). Results are given in **Figure 4**. Finally, the extended model predictive potential was additionally validated by *in silico* perturbation experiments with repetitive single-pulse changes in CRH and AVP concentrations both separately and conjointly. Appropriate results are presented in **Figure 5**.

**Table 2:** Oscillation amplitudes of cortisol (Ampl.) in 10^−8^ mol dm^−3^ for two fixed values of k_2.2_ and varied values of k_2.1_.

k_2.1_(10^−8^ mol dm^−3^ min^−1^)	k_2.2_ (10^−8^ mol dm^−3^ min^−1^)		
	0*	2.745	
	Ampl. for all k_5.3_	Ampl. for low k_5.3_	Ampl. for high k_5.3_
1.3725	0	0	0
1.4640	0	0.2400	0
1.5189	0	0.7340	0
1.5555	0	0.8845	0.3765
1.6470	0.5695	1.0100	0.8965
1.7385	0.9590	0.7645	1.0100
1.7751	1.0070	0.4400	0.9725
1.8300	0.9810	0	0.7970
1.8849	0.8075	0	0.2665
1.9215	0.5370	0	0
2.1960	0	0	0

*If k_4_ = 0, and [AVP]_0_ = 0, it corresponds to the initial HPA axis activity model (54).

Values of k_5.3_< 10^8^ mol^−1^ dm^3^ min^−1^ are considered as low k_5.3_, while k_5.3_ > 10^13^ mol^−1^ dm^3^ min^−1^ as high k_5.3_. A zero-amplitude value indicates the absence of oscillatory states, i.e. the presence of stable steady states in a denoted case.

**Table 3:** Oscillation amplitudes of cortisol concentrations (Ampl.) in 10^−8^ mol dm^−3^ for two fixed values of k_2.1_ and varied k_2.2_.

k_2.2_(10^−8^ mol dm^−3^ min^−1^)	k_2.1_ (10^−8^ mol dm^−3^ min^−1^)			
	1.83		1.00	
	Ampl. for low k_5.3_	Ampl. for high k_5.3_	Ampl. for low k_5.3_	Ampl. for high k_5.3_
0*	0.9810	0.9810	0	0
0.0183	0.9645	0.9810	0	0
0.1830	0.9440	0.9810	0	0
1.6470	0.4865	0.9810	0	0
1.7385	0.4205	0.9755	0	0
1.8300	0.3385	0.9660	0	0
2.2875	0	0.9010	0	0
2.7450	0	0.7970	0	0
3.2025	0	0.6350	0	0
3.6600	0	0.3385	0	0
3.7515	0	0.2230	0	0
3.8430	0	0	0	0
11.2000 (C1)	0	0	0	0
11.4000 (C2)	0	0	0.1550	0
17.6000 (C3)	0	0	0	0.7715
18.6000 (C4)	0	0	0	0
Higher	0	0	0	0

*If k_4_ = 0 and [AVP]_0_ = 0, it corresponds to the initial HPA axis activity model (54).

Values of k_5.3_< 10^8^ mol^−1^ dm^3^ min^−1^ are considered as low k_5.3_, while k_5.3_ > 10^13^ mol^−1^ dm^3^ min^−1^ as high k_5.3_. A zero-amplitude value indicates the absence of oscillatory states, i.e. the presence of stable steady states in a denoted case.

Moreover, let us know that the initial model described in (54) can be considered as the limiting case of the here considered model when the values of k_2.2_, k_4_ and initial concentration of AVP are all zero. In addition, in that case the value of the CRH inflow rate constant k_2.1_ in extended model ought to be the same as k_2_ value in the initial model, i.e. 1.83 × 10^−8^ mol dm^−3^ min^−1^. However, in order to get better insight in local behavior of the extended model, the value of k_2.1_ was varied in the range (1.3725 - 2.1960) × 10^−8^ mol dm^−3^ min^−1^ and k_2.2_ in the range (0.0183 - 6.4050) × 10^−8^ mol dm^−3^ min^−1^. Besides, for selected values of k_2.1_ and k_2.2_, the bifurcation diagrams with synergy constant (k_5.3_) as control parameter were analyzed using Method 2. Overall, positions of points with all types of dynamics identified by Method 2 are given in **Figure 2A**. Their actual nature qualitatively corresponds to one of the four forms of bifurcation diagrams presented in **Figure 2** B1 – B4. Results in **Figure 2** show that in points which lay far enough outside of the oscillatory domain (**Figure 1**), only stable steady states were obtained for all applied k_5.3_ values (**Figure 2**, B1 and all such cases are designated by **●**). In the case of points lying deep enough within the oscillatory domain in **Figure 1**, only oscillatory states exist for all applied k_5.3_ (**Figure 2**, B3 and all such cases are designated by ◼). It was observed that in this area, amplitude of oscillations depends on the value of k_5.3_ and this dependence was subjected to additional analysis. On the other hand, in points found in vicinity of both lower and upper borders of oscillatory domain in **Figure 1**, transitions from oscillatory dynamics into stable steady states and vice versa, respectively, were induced by varying the value of k_5.3_ (**Figure 2**, B2 and B4 and all such cases designated by ► and ◄, respectively).

A more detailed analysis of the influence of two inflow reaction constants k_2.1_, k_2.2_ and the synergy reaction constant k_5.3_ on the global behavior of the extended HPA model was done by comparing dynamic states between the bifurcation diagrams obtained for several fixed values of each of the inflow rate constant. In the cases where oscillatory dynamic states were identified, amplitudes and their periods were of particular interest for mutual diagrams comparison. In the whole range of applied changes obtained by varying all three constants, the oscillation periods varied in very narrow range between 20 min and 30 min. This variation of periods is even lower within one bifurcation diagram where oscillatory states occurred; in these cases, periods were rather constant or nearly constant, although k_5.3_ values were applied in extremely wide extent (from 1.098 × 10^3^ to 1.098 × 10^20^ mol^−1^ dm^3^ min^−1^) (data not shown).

On the other hand, the amplitude changes were much more diverse and interesting. Selected results are presented in **Tables 2**, **3**. Results in **Table 2** show variations of dynamic states and amplitude values of cortisol oscillations if k_2.1_ is varied for fixed k_2.2_ value. Two typical cases of k_2.2_ are given. In the first case k_2.2_, k_4_ and initial concentration of AVP are all equal to zero. This corresponds to the initial model (54). Under these conditions, amplitudes do not depend on k_5.3_ and hence only one value of amplitude is given for each k_2.1_ value. In this case, for lower values of k_2.1_ (such as for k_2.1_< 1.6470 × 10^−8^ mol dm^−3^ min^−1^) stable steady states were observed first. With k_2.1_ increase, HPA system passes to another stable steady state transiting through oscillatory region confined between two supercritical AH bifurcation points. It was also observed that the amplitudes reach their maximum at a certain k_2.1_ value within oscillatory region, which gradually decreases with approach to the AH points. In the second case, where k_2.2_ is non-zero, for 2.745 × 10^−8^ mol dm^−3^ min^−1^ (**Figure 2**), the dynamics is much more complex since dynamic state depends strongly on values of both k_5.3_ and k_2.1_. Namely, for low values of k_2.1_, only stable steady states were found as in the first case (**Figure 2**, **●**). With slightly increased k_2.1_ (such as for k_2.1_ = 1.4640 × 10^−8^ mol dm^−3^ min^−1^ in **Table 2** and **Figure 2**, ►) oscillatory dynamic states were found in region of low k_5.3_ values but stable steady states in the region of high k_5.3_ values. With further increase of k_2.1_, only oscillatory dynamic states are present for all k_5.3_ values. This refers to four values of k_2.1_ in **Table 2**, which correspond to four adjacent points denoted by ◼ in **Figure 2**. Moreover, the values of amplitude were found to be influenced by k_5.3_. At modestly low values of k_2.1_, oscillations with higher amplitudes were observed in the region of low k_5.3_ values, while smaller amplitudes were observed in the region of high k_5.3_ values (**Table 2**, for k_2.1_ = (1.5555 and 1.6470) × 10^−8^ mol dm^−3^ min^−1^). On the other hand, if values of k_2.1_ are modestly high (**Table 2**, for k_2.1_ = (1.7385 and 1.7751) × 10^−8^ mol dm^−3^ min^−1^), the k_5.3_ influence is reversed so that amplitudes are then positively correlated to k_5.3_. It could be noticed that in this middle k_2.1_ range, for the values of k_2.1_ such as (1.6470 or 1.7385) × 10^−8^ mol dm^−3^ min^−1^, the amplitudes are higher than the ones corresponding to the same values of k_2.1_ in the initial model (**Table 2**, k_2.2_ = 0), for all applied k_5.3_ values or for high k_5.3_, respectively. Further increase in k_2.1_, leads to the value of 1.83 × 10^−8^ mol dm^−3^ min^−1^ which corresponds exactly to k_2_ value in initial model published in previous paper (54). In this case, stable steady states and oscillatory states were found for low and for high k_5.3_ values, respectively (**Figure 2**, ◄). Finally, for the highest used values of k_2.1_, such as (1.9215 and 2.1960) × 10^−8^ mol dm^−3^ min^−1^ in **Table 2**, only stable steady states were observed in the whole k_5.3_ range. This corresponds to two adjacent points in **Figure 2**, depicted by **●**.

The observed changes in cortisol oscillation amplitudes when k_5.3_ is used as control parameter are localized in range of k_5.3_ values lying roughly between 10^8^ and 10^13^ mol^−1^ dm^3^ min^−1^ (See, for example, cases B1 – B4 in **Figure 2**), although this rate constant was varied in wide interval of values between 1.098 × 10^3^ and 1.098 × 10^20^ mol^−1^ dm^3^ min^−1^.

Relationships between dynamic states and amplitude values of cortisol oscillations when k_2.2_ is varied for fixed k_2.1_ value are shown in **Table 3**. Two typical cases of k_2.1_ were considered in the extended model. For the first case, k_2.1_ was chosen to be equal to 1.83 × 10^−8^ mol dm^−3^ min^−1^ so that the extended model could be appropriately compared with the initial model (54). The other case corresponding to k_2.1_ = 1.00 × 10^−8^ mol dm^−3^ min^−1^ is more general, providing conditions for a complete set of dynamic states similar to the ones obtained in bifurcation diagrams B1 – B4 (**Figure 2**). This case also corresponds to line C in **Figure 1** and is used as a representative example to explain the HPA system behavior near the bifurcation points (**Figure 3**).

Namely, the first case, where k_2.1_ is equal to 1.83 × 10^−8^ mol dm^−3^ min^−1^, shares the same rate constant value of the CRH inflow as the initial HPA model (54), for which the system is in the oscillatory state with amplitude of 0.981 × 10^−8^ mol dm^−3^ (as for k_2.2_ = 0, k_4_ = 0 and [AVP]_0_ = 0 in **Table 3** and point depicted by ◼ laying on the x-axis itself in **Figure 2**). For very low value of k_2.2_ such as 0.0183 × 10^−8^ mol dm^−3^ min^−1^, there is a weak to non-existent influence of k_5.3_. Oscillations are observed (**Figure 2**, ◼) and almost unchanged in the whole range of k_5.3_ values (**Table 3**). The contribution of AVP to system’s dynamics (if any) would probably originate predominantly from the magnocellular inflow source, since k_4_ has greater influence than k_2.2_ in the vicinity of x-axis (where k_2.2_ → 0). Yet, for the value of k_4_ equal to 1.537 × 10^−9^ mol dm^−3^ min^−1^ (**Table 1**), k_4_ is inferior to the impact of the rate constant of CRH inflow. Therefore, with k_5.3_ increase, system exhibit behavior similar to the behavior of the initial model. However, by increasing k_2.2_, the effect of k_5.3_ becomes noticeable. First, increasing the value of k_2.2_ leads to faster oscillation amplitudes decrease for low k_5.3_ compared to the high k_5.3_ values. With further k_2.2_ increase, system transits through AH bifurcation point and oscillatory states are replaced by stable steady states for low k_5.3_, while oscillations for high k_5.3_ continue to decrease (**Table 3** and **Figure 2**, five adjacent ◄). Finally, for sufficiently large k_2.2_, oscillatory states completely disappear for high k_5.3_ values. With any further k_2.2_ increase, k_5.3_ does not have any significant influence on HPA dynamics and only stable steady states could be observed for all k_5.3_ values. This corresponds to two adjacent points denoted by **●** in **Figure 2**.

In the other case, where k_2.1_ is equal to 1.00 × 10^−8^ mol dm^−3^ min^−1^, the HPA system is in stable steady states for the lowest k_2.2_ values, unlike in the first case. By increasing the k_2.2_, the system passes through the oscillatory domain *via* two AH bifurcations and finally enters to other stable steady states for higher k_2.2_ values (**Figure 1**). This value of k_2.1_ belongs to the points C1 - C4 region in **Figure 1**. The global behavior of the system in the vicinity of these two AH bifurcations was examined in these four points on line C which are listed in **Table 3**. Two of them (C1 and C2) are very close to the first (lower) AH bifurcation and the other two (C3 and C4) are very close to the second one (upper). Obviously, the points were chosen to cover both sides of bifurcation points. Namely, for the lowest k_2.2_ value (11.20 × 10^−8^ mol dm^−3^ min^−1^), corresponding to the point found below the first (lower) AH bifurcation (**Figure 1**, C1), only stable steady states were detected for all k_5.3_ values. For the nearby value of the k_2.2_ which is on the opposite side of this AH bifurcation (**Figure 1**, C2), oscillations were detected for low k_5.3_ values, but only stable steady states for high k_5.3_ values. On the other hand, by increasing the k_2.2_ to the value slightly below the second (upper) AH bifurcation (**Figure 1**, C3), dynamic states were inversed so that oscillatory states were then observed only for high k_5.3_ values, while for low k_5.3_ values, only stable steady states were present. For the nearby value of k_2.2_ (18.60 × 10^−8^ mol dm^−3^ min^−1^) which is a bit above this AH bifurcation (**Figure 1**, C4), stable steady states were detected in the whole range of applied k_5.3_ values. Described four bifurcation diagrams for points C1-C4 obtained by Method 1b and Method 2 are shown in **Figure 3**.

Both Method 1b and Method 2, provide mutually consistent results in a sense that oscillations are obtained when steady state is unstable and oscillations are absent if steady state is stable. From **Figure 3**, it may be noticed that change of cortisol concentrations during the transition from one steady state to another in points C1 and C4, is localized within relatively narrow and middle interval of k_5.3_ values close to the positions of the AH bifurcation points observed in points C2 and C3. At the same time, in vicinity of AH bifurcation points, cortisol steady-state concentrations are gradually changed. The above described behavior presented in **Figure 3** can also be found in bifurcation diagrams B1 – B4 presented in **Figure 2** with the similar k_5.3_ interval. Additionally, change of amplitude of cortisol oscillations in bifurcation diagram B3 in **Figure 2** is confined within the same range of k_5.3_ values. Out of this range, for k_5.3_< 10^8^ mol^−1^ dm^3^ min^−1^ and k_5.3_ > 10^13^ mol^−1^ dm^3^ min^−1^, that correspond to low and high k_5.3_ values, respectively, dynamic states are almost independent on k_5.3_. Described global behavior of the system is the result of investigations in points on line C very close to the boundaries of the oscillatory domain obtained by Method 1a for k_5.3_ = 3.66 × 10^2^ mol^−1^ dm^3^ min^−1^ (**Table 1**). If oscillatory domain is obtained for different k_5.3_ value, some of these points, if not all, (including the ones on A, B, D and E lines) may be positioned differently relative to the new borders of oscillatory domain. This could provide an additional insight into the interpretation of corresponding bifurcation diagrams in **Figures 3**, **2**.

In order to further correlate obtained results with experimental findings in the literature, the examinations of mutual combinations of k_2.1_ and k_2.2_ values for which system transits into oscillatory states of higher amplitudes with increase of control parameter (k_5.3_) were of particular interest. Three types of distinct changes were found by comparing how amplitude of oscillations increases with control parameter in the initial and extended model when their CRH inflow is the same. They will be discussed in more details using the examples of points P, Q and R in **Figure 2**: point P (k_2.1_ = k_2.2_ = 1.830 × 10^−8^ mol dm^−3^ min^−1^), point Q (k_2.1_ = 1.738 × 10^−8^ mol dm^−3^ min^−1^, k_2.2_ = 2.745 × 10^−8^ mol dm^−3^ min^−1^) and point R (k_2.1_ = 1.647 × 10^−8^ mol dm^−3^ min^−1^, k_2.2_ = 4.575 × 10^−8^ mol dm^−3^ min^−1^). In **Figure 4**, the bifurcation diagrams ((P1) - (R1), ◈), and diagrams of variation of [CORT] oscillation amplitudes ((P2) - (R2), ⦿) in all three points, together with the samples of temporal evolutions of [CORT] for k_5.3_ = 1.098 × 10^16^ mol^−1^ dm^3^ min^−1^ ((P3) - (R3), thinner curves) in an arbitrarily selected time interval are presented. Results referring to the initial model (54) for the same values of the rate constant of CRH inflow as in cases P, Q and R, are incorporated into corresponding diagrams in **Figure 4** and depicted by symbols ◆, **●** and thicker curves. Namely, the increase of [CORT] amplitudes with k_5.3_ increase can be clearly seen in all three cases P, Q and R in diagrams (P1) - (R1) (◈) and (P2) - (R2) (⦿). However, it could be noticed that in the case P, oscillation amplitudes of [CORT] in extended model are lower than the ones in the initial model in the whole range of applied k_5.3_ [**Figure 4**, (P2)]. Inversely, in the case R, oscillation amplitudes of [CORT] are higher in the extended model than the one in initial model for all applied k_5.3_ [**Figure 4**, (R2)] and could be about 1.3- to 2-fold greater. On the other hand, for a certain combination of k_2.1_ and k_2.2_ values, [CORT] amplitudes in extended model could exceed the one corresponding to the initial model, but only for high k_5.3_ values, as indicated in the case Q [**Figure 4**, (Q2)]. The described [CORT] amplitude differences between two models in all three cases could also be directly observed in **Figure 4**, (P3) – (R3) for k_5.3_ = 1.098 × 10^16^ mol^−1^ dm^3^ min^−1^. Due to the indications of conditions in the extended model under which amplitude of [CORT] ultradian oscillations may increase due to synergistic effect of CRH and AVP on stimulating ACTH secretion by corticotrope cells, the extended model for point R was subjected to further analysis.

Hence, *in silico* perturbation experiments were performed to additionally assess the predictive potential of the extended model in the conditions corresponding to point R (**Figure 2**) and k_5.3_ = 1.098 × 10^10^ mol^−1^ dm^3^ min^−1^ (a value around the middle in **Figure 4**, (R1) and (R2)). The perturbations were induced by repeating single-pulse changes in CRH and AVP concentrations applied both separately and conjointly, and which were consistent in intensity and time of application. Results are presented in **Figure 5** and show the apparent influence of acute changes in CRH or/and AVP levels on global ultradian dynamics of the HPA axis model.

As expected, separate perturbations with CRH and AVP elicited a quantitatively similar response of the HPA axis extended model, only if applied concentration (perturbation intensity) of AVP was order of magnitude higher compared to CRH (**Figures 5B, C**). Also, a synergistic effect is observed in the changes of the cortisol oscillation amplitudes in response to their concurrent perturbations (**Figure 5D**). The pattern of the system response to this perturbation is qualitatively similar to the one induced by perturbations with CRH solely. Moreover, as expected from our previous experience (54, 62), perturbations caused a highly variable response depending on the phase angle of the selected ultradian oscillation at which the first perturbation was applied. Therefore, even uniform periodic perturbations with small intensities induced strongly irregular (stochastic) appearances of [ACTH] (not shown) and [CORT] oscillations.

## Discussions

According to presented results, various ultradian oscillatory dynamics can be achieved, depending on the individual values of CRH and AVP inflows, as well as the rate constant of their synergistic reaction. For a particular combination of the values of inflows rate constants used in this study, there are values of synergy rate constant k_5.3_ that could influence the HPA system to enter into the oscillatory dynamic states from stable steady state and vice versa. It has been found that transitions between these dynamic states were always through supercritical AH bifurcation point.

Due to the features of the supercritical AH bifurcation, some of the well-documented properties of the HPA axis activity could be more plausibly reproduced. For instance, elasticity of HPA axis in adjusting its dynamics in order to maintain homeostasis under the action of stressful stimuli can be simulated by reversible transitions between stable steady and oscillatory dynamic states in the vicinity of supercritical AH bifurcation. These dynamic transitions are characterized by gradual decrease in ultradian oscillation amplitudes as the system approaches supercritical AH bifurcation, causing the system to exhibit growing response to the same perturbation intensity.

It should be pointed out that above described qualitative dynamic transitions in HPA axis activity under the influence of synergy rate constant are related to the system’s global behavior in points close to the boundaries of the oscillatory domain obtained by Method 1a for the value of k_5.3_ in **Table 1** (**Figure 1**). For oscillatory domain obtained for different k_5.3_ value, some of these points, if not all, may be positioned differently relative to the new borders of oscillatory domain. This could provide rather additional insight into the interpretation of their bifurcation diagrams in **Figure 3**, but in **Figure 2** as well.

On the other hand, for examined points lying deep enough within the oscillatory domain, much more diverse quantitative changes in HPA activity were identified in corresponding bifurcation diagrams. Comparison of the results of bifurcation analyses for points in which amplitude of cortisol oscillations increases with k_5.3_ in the initial and extended model for the same CRH inflow constant, revealed that extended model is capable to provide potential conditions under which ultradian amplitudes of cortisol concentrations could increase several-fold due to CRH and AVP synergistic action on corticotrope cells. This is in agreement with experimental observations in studies conducted on humans during stress (39, 44–47).

The potential of the extended model under selected conditions to anticipate a synergistic effect in the HPA axis ultradian dynamics response was verified in *in silico* experiments with perturbations induced by repeating single-pulse changes in CRH and AVP concentrations applied both separately and simultaneously (**Figure 5**). The observed qualitative similarity between response patterns of the HPA axis to perturbation with CRH solely and conjointly with AVP (**Figures 5B, D**) can be also found between the results of experiments in humans with 10-hour infusions of CRH and of both peptides simultaneously (39). Although cortisol oscillations obtained by the proposed model are extremely regular (both in period and amplitude) (**Figure 5A**), the ones obtained after series of single-pulse perturbations that were regular in intensity and time of application, are irregular (chaotic) (**Figures 5B–D**). They actually more resemble experimentally measured cortisol level fluctuations frequently encountered in literature (4, 8, 9, 18, 21–23, 38, 55, 63–65), while cortisol oscillations obtained by the extended model under conditions that may be regarded as ideal for the HPA axis (i.e. without any perturbation over the time whatsoever). Moreover, the sensitivity of an oscillatory dynamics depends strongly on the phase angle of the selected ultradian oscillation at which the first perturbation was applied and thus cortisol oscillations amplitude may decrease, increase or remain unaltered. Nevertheless, further *in silico* examinations of the proposed extended model under these and similar conditions are required to fully address stress-related effects of AVP and CRH on HPA ultradian dynamics reported in the literature.

Furthermore, the extended model simulated the experimentally observed inferiority of AVP as an ACTH secretion stimulator compared to CRH in humans. Namely, both in human (39, 43–47, 55) and in rat (26, 36–38, 40, 41), CRH reaction pathway is considered to be dominant in pituitary-adrenal regulation. There are results in several studies *in vitro* in rat anterior pituitary cells and *in vivo* in rats, indicating the necessity for much higher concentrations of exogenously administered AVP in order to induce similar effects on ACTH secretion as certain injected CRH concentrations (37, 38, 40, 41). In line with above mentioned experimental findings, results in **Figure 1** show that a much larger (circa by an order of magnitude) increase in the values of parvocellular AVP inflow source (k_2.2_) is required to retain HPA model in the oscillatory regime after a small decrease in the value of parvocellular CRH inflow source (k_2.1_). Additionally, an order of magnitude higher AVP perturbation intensity (AVP concentration) was needed to induce quantitatively similar response of HPA axis model to the one elicited by perturbation with CRH solely (**Figures 5B, C**). Moreover, the extended model predicted the CRH reaction pathway to almost completely prevail over the AVP reaction pathway under certain condition (**Figures 1**, **2**). However, for some other conditions given in **Figure 1**, the extended model also predicted the feasibility of AVP reaction pathway governed by parvocellular AVP inflow source to take over the CRH supremacy (**Figure 1**). In similar conditions, the possibility for the HPA axis to exhibit oscillatory dynamics for negligible amounts of CRH was shown as well.

Additionally, during stress, particularly chronic stress and after adrenalectomy, the number of parvocellular CRH neurosecretory cells that co-produce AVP was found to increase considerably as do the amounts of synthesized AVP compared to CRH *per* cell (66, 67). Very good agreement with these experimental findings was obtained in simulations using the extended model proposed here, where by increasing the k_2.2_ value much more than the value of k_2.1_, higher AVP to CRH concentration ratios could be yielded (**Figure 1**).

At the same time, during all the analyses conducted in this study, oscillating nature of dynamic state, and even frequency of cortisol ultradian oscillations has been found relatively sustained under all investigated conditions. This is in line with experimental findings indicating preserved endogenous HPA pulsing system in humans under various conditions (6, 9, 39).

Although, the HPA axis activity model proposed here is capable to provide good results, it however also inherited several limitations. The most notable limitation stems from the conciseness of the model where many complex processes were represented by summarized and simplified reaction steps (R1) - (R15) in **Table 1**. Thereby, since the peptide precursors of ACTH and other steroid hormones were not included in the initial model, nor in the current extended model, concentration of ACTH was several orders beyond its reference range values. This discrepancy may be corrected by further model augmentation with introduction of lacking reaction species and their relations, in a similar way as it was done with preceding models (54, 68, 69, for instance) that were developed from our core model (58). The same “strategy” would be applied if a certain process or the influence of a certain bioactive substance ought to be examined in more details in regard to its role in HPA axis activity, such as: cAMP/PKA, PLC/IP(3), DAG/PKC signal transduction pathways, gene transcription and translation, epinephrine (adrenalin), angiotensin II, etc. On the other hand, the low-dimensional models can be more easily manipulated mathematically with aim to define desirable dynamic states, before being extended for different applications. This is usually not the case with more detailed non-stoichiometric models.

Based on the results presented in this study, stoichiometric modeling approach, bifurcation analyses and numerical simulations have proven to be very helpful in comprehending the complex involvement of AVP in HPA axis ultradian dynamics. The proposed model provides a good basis for further investigation of conditions in which the particularly amplified effect of CRH on corticotrope cells by magnocellular AVP can be of vital importance. These conditions are associated with life-threatening circumstances such as the risk of hyponatremia due to severe hypovolemia that can occur during arduous physical work or those requiring long-term sustained rise of adrenal corticosteroids due to their known immunosuppressant and anti-inflammatory effects. Furthermore, since it is known that “AVP regulates ACTH release under certain conditions, and exogenously administered AVP is used clinically to stimulate ACTH secretion” (70) as well as that considered process is very complex for experimental investigations, the proposed model with related numerical simulations can be obviously useful for determining the appropriate drug dose for therapeutic purposes.

## Conclusions

The proposed extended HPA model is positively correlated with several experimental findings in the literature and offers the potential to proceed *in silico* investigations of the influence of AVP and its synergistic action with CRH on the HPA axis dynamics. Expanding the pre-existing initial HPA model with AVP contributes to the enhancement of the model’s comprehensiveness and biological plausibility, while still being sufficiently tractable to mathematical analysis and numerical simulation, despite the increased number of dynamic variables. Moreover, the presented model provides a good basis for its further development and adjustments to align with experimental finding under physiologically normal as well as various stressful conditions. Further refinement of the present model seems to be necessary if hormone levels should be quantitatively compared with experimental measurements and if apparently stochastic form of oscillations would be the aim of some future study.

## Data availability statement

The original contributions presented in the study are included in the article/**Supplementary Material**. Further inquiries can be directed to the corresponding authors.

## Author contributions

ASS contributed to conception of the study, extended model development and adjustments, conducting the analysis, results processing and interpretation, manuscript writing. ŽČ wrote the first manuscript draft, contributed to extended model development and adjustments, study design, organizing the results and interpretation. SM contributed to conducting the analysis and results processing. AI-Š also contributed to conducting the analysis, the results processing and interpretation. LjK-A contributed to study design, extended model development and adjustments, to results organization and interpretation. All authors listed have made a substantial, direct and intellectual contribution to the work, manuscript revision and approved submitted version.

## Funding

We are thankful for the financial support from the Ministry of Sciences and Technology of Republic of Serbia (Grant Numbers 172015 and 45001, and Contract numbers: 451-03-68/2022-14/200026, 451-03-68/2022-14/200146 and 451-03-68/2022-14/200051. This research was also supported by Science Fund of Republic of Serbia #Grant Number. 7743504, *Physicochemical aspects of rhythmicity in NeuroEndocrine Systems: Dynamic and kinetic investigations of underlying reaction networks and their main compounds*, NES.

## Acknowledgments

We are grateful to Dr. Ana Stanojević, a formerly employee of the Faculty of Physical Chemistry, University of Belgrade, who was working with us at the beginning of these investigations.

## Conflict of interest

The authors declare that the research was conducted in the absence of any commercial or financial relationships that could be construed as a potential conflict of interest.

## Publisher’s note

All claims expressed in this article are solely those of the authors and do not necessarily represent those of their affiliated organizations, or those of the publisher, the editors and the reviewers. Any product that may be evaluated in this article, or claim that may be made by its manufacturer, is not guaranteed or endorsed by the publisher.
