# Supplementary material for: Influence of arginine vasopressin on the ultradian dynamics of Hypothalamic-Pituitary-Adrenal axis
Source: Front Endocrinol (Lausanne). 2022 Oct 5;13:976323. doi: 10.3389/fendo.2022.976323 (PMC9581400; doi:10.3389/fendo.2022.976323)
Supplement: Supplementary file 1 [file DataSheet_1.docx]

The illustration of Method 1a, Method 1b and Method 2

In order to examine dynamics of HPA axis model, the bifurcation analysis was applied. It was performed by using two approaches. The first approach based on the methods of numerical continuation (Methods 1a and 1b) and the second one based on numerical simulations of dynamic states obtained by the differential equations (1) - (6). In all cases the goal was to determine boundaries of oscillatory domain of the model of HPA axis.

**Method 1a** - In this approach we analyzed how position of Andronov-Hopf (AH) bifurcation change at different values of k_2.1_ when k_2.2_ is used as bifurcation parameter. Thus, the value of k_2.1_ was varied in the range 1.8 × 10^−9^ < k_2.1_ < 1.8 × 10^−8^ mol dm^−3^ min^−1^. At each selected value of k_2.1_ bifurcation analysis using the methods of numerical continuation with k_2.2_ as bifurcation parameter was done by changing its value. For each value of k_2.2_ steady-state concentrations were calculated and Hurwitz determinants were evaluated in order to test stability of steady-state and to check if the emergence of AH bifurcation occurred. An example of the performed calculation is presented in Figure S1 (a). In this case the value of k_2.1_ was set to be 1.8 × 10^−8^ mol dm^−3^ min^−1^ while the value of k_2.2_ was varied in the range 5.0 × 10^−8^ ≤ k_2.2_ ≤ 2.0 × 10^−7^ mol dm^−3^ min^−1^. Thin solid line represents stable steady states while bold solid line represents region between AH bifurcations where oscillatory domain exists.





**Figure S1.** Bifurcation diagrams obtained using methods of numerical continuation for cases: (a) Method 1a, (b) Method 1b; thin solid line represents stable steady states while bold solid line represents oscillatory region.

**Method 1b** - In this case for different selected values of k_2.1_ and k_2.2_ bifurcation analysis with k_5.3_ as bifurcation parameter was carried out by using methods of numerical continuation. During bifurcation analysis only value of k_5.3_ was varied while the values of remaining parameters were kept at constant values. Example of this analysis with the values k_2.1_ = 1.0 × 10^−8^ mol dm^−3^ min^−1^ and k_2.2_ = 17.7 × 10^−8^ mol dm^−3^ min^−1^ is presented in Figure S1 (b).

**Method 2** - Here, the influence of control parameter k_5.3_ on the selected dynamic states realized when k_2.1_ and k_2.2_ had the following values: 1.0 and 17.7 × 10^−8^ mol dm^−3^ min^−1^, respectively, was examined. Namely, for each value of the control parameter k_5.3_, numerical simulations were performed. If the stable steady states were occurred, the concentration of cortisol ([CORT]) in that state was presented in the bifurcation diagram by one point (Figure S2). The oscillatory dynamic state was presented by two points denoting maximum and minimum of characteristic oscillation under these conditions (Figure S2).





**Figure S2.** The bifurcation diagram that presents the influence of control parameter k_5.3_ on the selected dynamic states realized when k_2.1_ and k_2.2_ had the following values: 1.0 and 17.7 × 10^−8^ mol dm^−3^ min^−1^, respectively (left side). On the right side, two different dynamic states are presented, for the arbitrarily chosen time interval between around 92 and 94 hours. (a) Stable steady state realized when k_5.3_ = 1.098 × 10^4^ mol^−1^ dm^3^ min^−1^, and (b) The oscillatory dynamic state when k_5.3_ = 1.098 × 10^14^ mol^−1^ dm^3^ min^−1^. Obviously, the oscillatory dynamic state was presented by two points denoting maximum and minimum of characteristic oscillation under these conditions.
